# Suppression of mRNAs encoding CD63 family tetraspanins from the carcinogenic liver fluke *Opisthorchis viverrini* results in distinct tegument phenotypes

**DOI:** 10.1038/s41598-017-13527-5

**Published:** 2017-10-30

**Authors:** Sujittra Chaiyadet, Watchara Krueajampa, Wiphawi Hipkaeo, Yada Plosan, Supawadee Piratae, Javier Sotillo, Michael Smout, Banchob Sripa, Paul J. Brindley, Alex Loukas, Thewarach Laha

**Affiliations:** 1 Department of Parasitology, Faculty of Medicine, Khon Kaen University, Khon Kaen, Thailand; 2 Electron microscopy Laboratory, Department of Anatomy, Faculty of Medicine, Khon Kaen University, Khon Kaen, Thailand; 3 Office of Academic Affairs, Faculty of Veterinary Sciences, Mahasarakham University, Mahasarakham, Thailand; 4 Centre for Biodiscovery and Molecular Development of Therapeutics, Australian Institute of Tropical Health and Medicine, James Cook University, Cairns, QLD, Australia; 5 Department of Pathology, Faculty of Medicine, Khon Kaen University, Khon Kaen, Thailand; 6 Department of Microbiology, Immunology and Tropical Medicine, and Research Center for Neglected Diseases of Poverty, School of Medicine & Health Sciences, George Washington University, Washington, DC 20037 USA

## Abstract

The liver fluke *Opisthorchis viverrini* infects 10 million people in Southeast Asia and causes cholangiocarcinoma (CCA). Fluke secreted and tegumental proteins contribute to the generation of a tumorigenic environment and are targets for drug and vaccine-based control measures. Herein, we identified two tetraspanins belonging to the CD63 family (*Ov*-TSP-2 and *Ov*-TSP-3) that are abundantly expressed in the tegument proteome of *O. viverrini*. *Ov-tsp-2* and *tsp-*3 transcripts were detected in all developmental stages of *O. viverrini*. Protein fragments corresponding to the large extracellular loop (LEL) of each TSP were produced in recombinant form and antibodies were raised in rabbits. *Ov*-TSP-2 and TSP-3 were detected in whole worm extracts and excretory/secretory products of *O. viverrini* and reacted with sera from infected hamsters and humans. Antibodies confirmed localization of *Ov*-TSP-2 and TSP-3 to the adult fluke tegument. Using RNA interference, *Ov-tsp-2* and *tsp-3* mRNA expression was significantly suppressed for up to 21 days *in vitro*. Ultrastructural observation of *tsp-2* and *tsp-3* dsRNA-treated flukes resulted in phenotypes with increased tegument thickness, increased vacuolation (*tsp-2*) and reduced electron density (*tsp-3*). These studies confirm the importance of CD63 family tegument tetraspanins in parasitic flukes and support efforts to target these proteins for vaccine development.

## Introduction

Liver fluke infection caused by *Opisthorchis viverrini* remains a serious public health problem in Southeast Asia^1^. Infection with *O. viverrini* has been associated with bile duct cancer, and the International Agency for Research on Cancer (IARC) designated the liver fluke as a class 1 carcinogen^2^. To date, control strategies for *O. viverrini* infection rely on drug therapy and health education, but this approach is not sustainable, and the development of a vaccine against *O. viverrini* infection would be a major advance for prevention of the infection.

The adult stage of *O. viverrini* resides in the bile ducts of the host where it secretes protein antigens that stimulate immunopathology^3^. *Opisthorchis viverrini* excretory-secretory (*Ov*-ES) products stimulate inflammation of the host bile duct epithelium and increase bile duct cell proliferation^4^. The fluke outer surface, called the tegument, is a dynamic host-interactive layer involved in nutrition, immune evasion and modulation, excretion, sensory reception and signal transduction, and tegumental proteins are major components of secretory products actively released by the flukes. Many different proteins are present in the tegument of *O. viverrini*, of which tetraspanins are major components^5^. Tetraspanins is a superfamily of membrane proteins characterized by the presence of four transmembrane domains and a large extracellular loop (LEL). They contribute to the maintenance of tegument membrane integrity^6^ and play a key role in tegument formation in parasitic flukes^7,8^. Previous studies identified the tetraspanin *Ov*-TSP-1 from the CD9/81 family, and its function in tegument integrity was highlighted by gene silencing approaches^8^. Several tetraspanins have been found in the tegument proteome and extracellular vesicles released from *O. viverrini* ^9,10^, and, in other trematodes, they are being tested as potential vaccine candidates^11,12^.

Herein, we identified and characterized two tetraspanin-encoding cDNAs designated *Ov-tsp-2* and *Ov-tsp-3* and investigated the impact of silencing their expression on tegument architecture in adult flukes. Understanding the functions of tetraspanins in *O. viverrini* will facilitate efforts to develop a vaccine against this carcinogenic fluke.

## Results

### General characteristics of *Ov-tsp-2* and *tsp-3* cDNAs and predicted proteins

**Figure 1:**
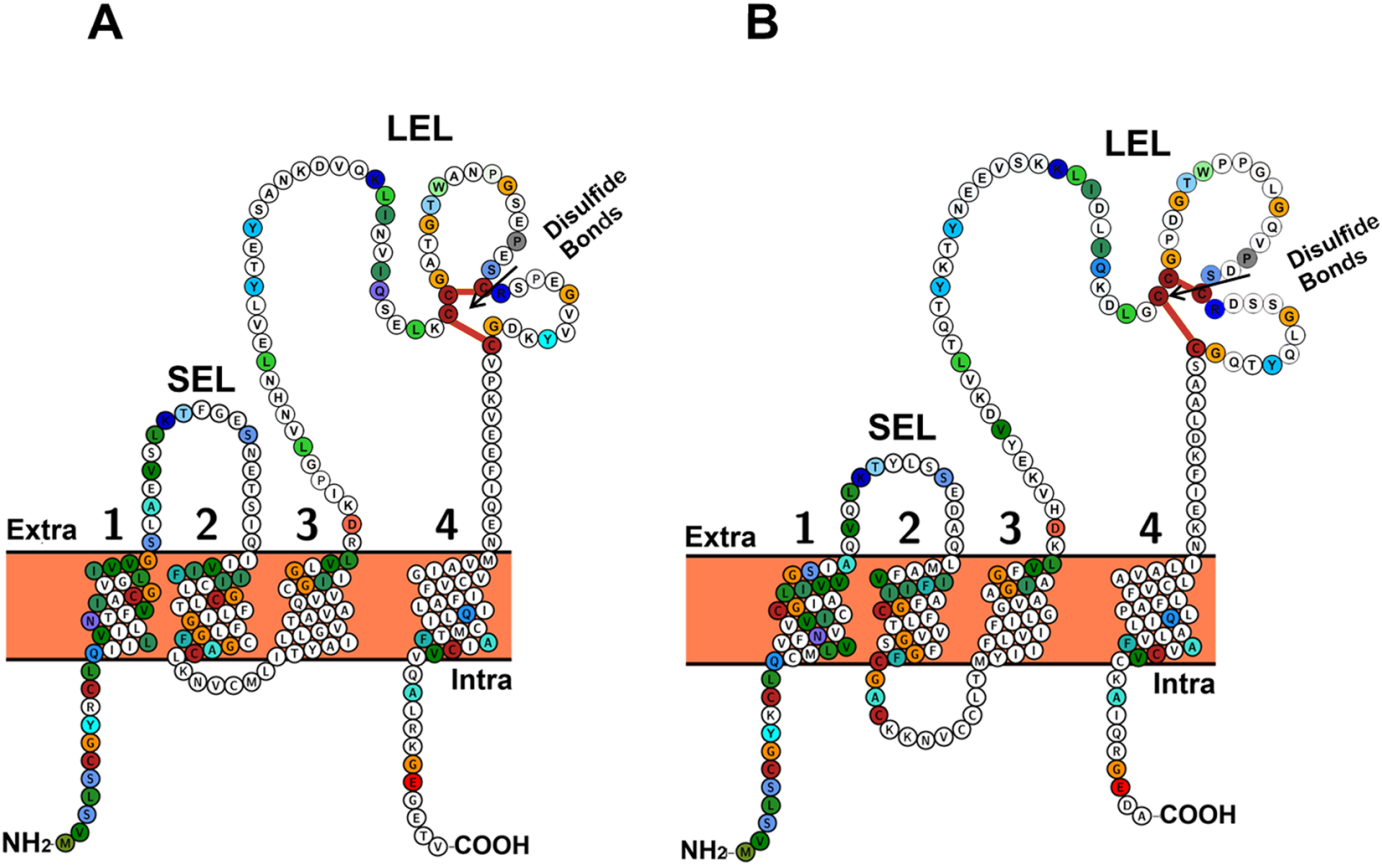
Predicted membrane spanning architecture of *Ov*-TSP-2 and TSP-3. Schematic illustration of the structure of *Ov*-TSP-2 (**A**) and *Ov*-TSP-3 (**B**) proteins visualized by Protter. The four membrane spanning domains are highlighted: small extracellular loop (SEL), large extracellular loop (LEL) with three conserved motifs (CCG, -PXSC and GC motifs), a small inner loop (IL) and short cytoplasmic tail are featured. The sequences labeled in colour indicate identity of amino acids between *Ov*-TSP-2 and *Ov*-TSP-3.

The full-length cDNA sequence of *Ov-tsp-2* was 672 base pairs, which encoded for a predicted protein of 223 amino acids and a molecular weight of 23.8 kDa. *Ov*-*tsp-2* contained four transmembrane domains consisting of a small extracellular loop (SEL; residues 34–52), a larger extracellular loop (LEL; residues 109–186), a small inner loop (IL; residues 79–84), and short cytoplasmic tail (residues 213–223). The LEL region of *Ov*-TSP-2 contained the conserved CCG motif and the -PXSC and GC sequences, which form disulfide bonds (Fig. 1A).

*Ov*-TSP-3 contains 666 base pairs encoding for a putative protein of 221 amino acids and 23.7 kDa, including a SEL (residues 37–50), LEL (residues 109–186), IL (residues 74–85) and a short cytoplasmic tail (residues 213–221) (Fig. 1B). *Ov*-TSP-3 also contains a CCG motif and PXSC and GC conserved sequences identical to those found in *Ov*-TSP-2 (Supplementary Fig. S1). Sequence homologies and similarities between *Ov*-TSP-2 and TSP-3 open reading frame were 46% and 65%, respectively. While LEL of *Ov*-TSP-2 and TSP-3 showed sequence homologies and similarities at 39% and 59%, respectively.

### Phylogeny of *O. viverrini* tetraspanins

**Figure 2:**
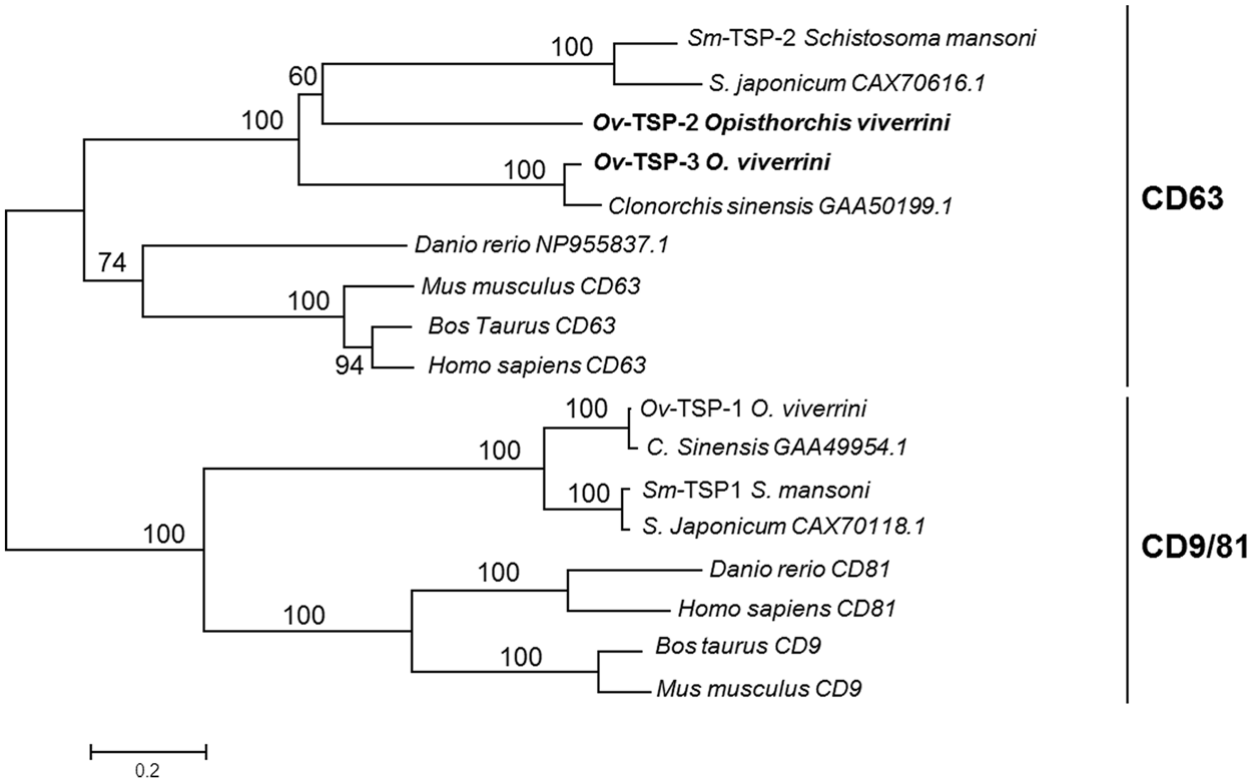
Neighbor joining phylogenetic tree of *Opisthorchis viverrini* tetraspanins and homologs belonging to the CD63 and CD9/81 lineages. Unrooted phylogenetic tree based on complete open reading frames. *Ov*-TSP-2 and TSP-3 grouped within the same clade as other CD63-like plathyhelminth sequences. The tree was calculated using 1,000 bootstrap samplings and values are shown on each branch.

*Ov*-TSP-2 and *Ov*-TSP-3 clustered together in the CD63 lineage of tetraspanins (Fig. 2). *Ov*-TSP-2 grouped with the *S. mansoni* vaccine antigen (*Sm*-TSP-2)^11,13^ and *S. japonicum* CAX70616.1, whereas *Ov*-TSP-3 fell in the sister group with *C. sinensis* GAA50199.1. Both clusters of flatworm CD63-like proteins formed a single clade that was distinct from the vertebrate CD63 proteins (Fig. 2). The previously described tetraspanin from *O. viverrini*, *Ov*-TSP-1^8^ grouped in the largest cluster of the tetraspanin family, the CD9 lineage^14^. The hallmark characteristics of CD9 and CD63 lineages are unclear, however our results revealed that the number of conserved cysteine residues in the LEL region is not sufficient to distinguish both families. From our constructed tree, the LEL of invertebrates (*O. viverrini*, *C. sinensis* and *Schistosoma* spp.) in the CD9 family has 6 cysteine residues, while vertebrate homologues (*D. rerio*, *B. taurus*, *M. musculus* and *H. sapiens*) have 4 cysteines in the LEL. In contrast, in the CD63 family, the LEL region of invertebrates has 4 cysteine residues (5 residues in *C. sinensis*) but vertebrate homologues have 6 cysteines. In the CD63 family, the three motifs (CCG, PXSC and GC) were 100% conserved. In contrast, while CD9 members do not have a conserved PXSC motif, they do show 100% conservation of the CCG and GC motifs.

### ***Ov***-TSP-2 and TSP-3 are immunogenic components of *O. viverrini* excretory/secretory products

**Figure 3:**
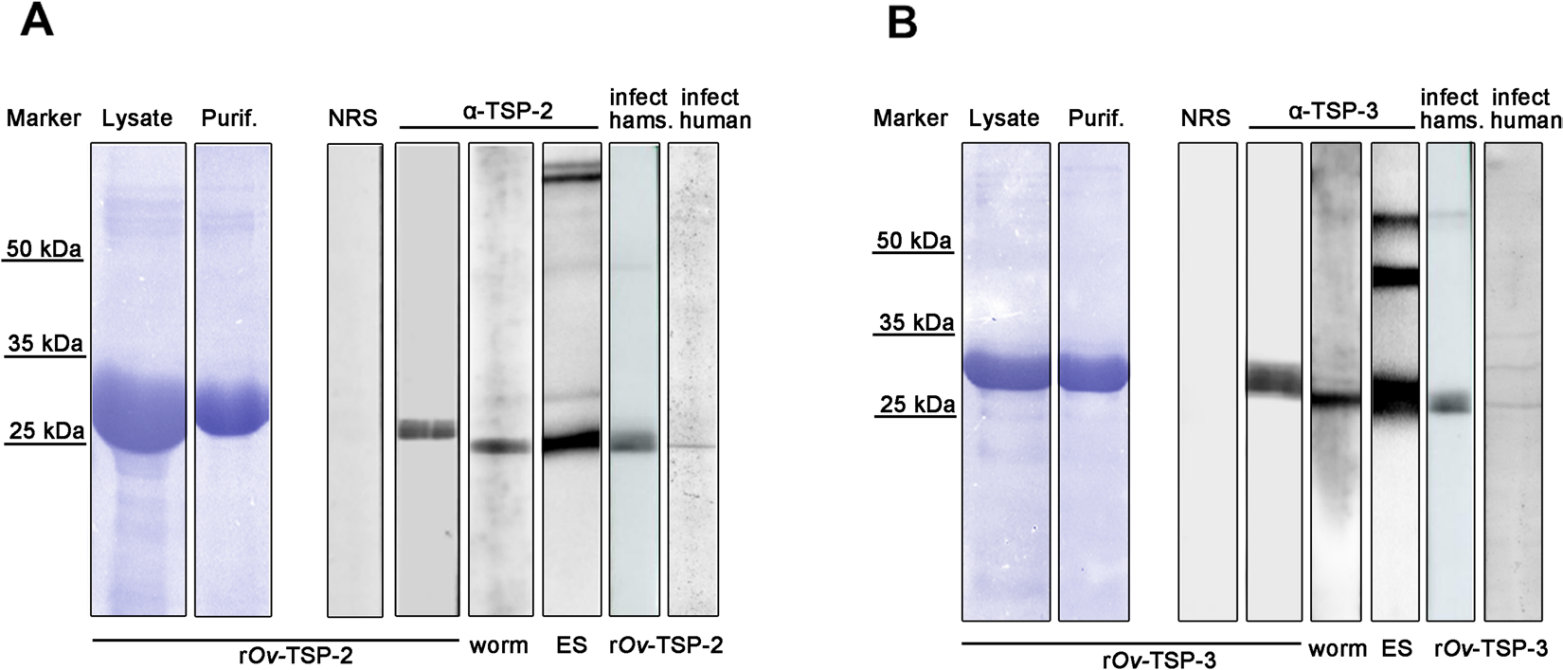
Recombinant *Ov*-TSP-2 and TSP-3 were expressed as thioredoxin fusion proteins in *E. coli* and were immunologically detected by sera from liver fluke infected hamsters and human subjects. The expressed and purified recombinant *Ov*-TSP-2 (**A**) and *Ov*-TSP-3 (**B**) proteins were separated using SDS-PAGE. SDS-PAGE of *E. coli* lysate (Lysate), and, purified protein using Ni-NTA affinity (Purif.) are shown. Western blot analysis of recombinant *Ov*-TSP-2 (r*Ov*-TSP-2) and *Ov*-TSP-3 proteins (r*Ov*-TSP-3), immunoblot of recombinant proteins probed with pre-immunization hamster serum (NSR); and immunoblot of recombinant proteins probed with rabbit anti-r*Ov*-TSP-2 or r*Ov*-TSP-3 (α-TSP-2 and α-TSP-3). *Ov*-TSP-2 or *Ov*-TSP-3 in crude worm protein extracts (worm) and in excretory/secretory products (ES) of *O. viverrini* were probed with antibodies against r*Ov*-TSP-2 or r*Ov*-TSP-3. Sera from liver fluke infected humans and hamsters recognize r*Ov*-TSP-2 and r*Ov*-TSP-3 (infect human and infect hams, respectively). [In panels A and B, the two lanes at the left of each labeled Lysate and Purif. were cropped (with Adobe Photoshop) from images of the entire gels; specifically lanes Lys and F3 for both panels A and B. Supplementary Fig. S2 presents the full length, intact gels.]

The recombinant LEL domains of *Ov*-*tsp-2* and *Ov*-*tsp-3* were cloned into the pET32a vector. *Ov*-TSP-2 and *Ov*-TSP-3 were expressed in *E. coli* as fusion proteins with an N-terminal thioredoxin (TRX)-His6-EK protease tag. The masses of the expressed proteins were 29.02 kDa (19.37 kDa TRX plus 9.65 kDa) for *Ov*-TSP-2 (Fig. 3A) and 28.82 kDa (19.37 kDa TRX plus 9.45 kDa) for *Ov*-TSP-3 (Fig. 3B).

The recombinant *Ov*-TSP-2 (r*Ov*-TSP-2) and *Ov*-TSP-3 (r*Ov*-TSP-3) proteins were probed with antibodies against both proteins generated in rabbits. Both r*Ov*-TSP-2 and r*Ov*-TSP-3 were identified by Western blot at their predicted molecular weights (~29 kDa –the LEL plus TRX) (Fig. 3A and B), and were not detected by pre-immunization serum, NSR (Fig. 3A and B), nor did they cross-react with antibodies against *E. coli* TRX (not shown). Antibodies against *Ov*-TSP-2 and *Ov*-TSP-3 were used to detect both proteins in whole fluke extract and ES products of adult *O. viverrini*. Moreover, recombinant *Ov*-TSP-2 and TSP-3 were recognized by sera from experimentally infected hamsters and naturally infected human subjects from an endemic area in northeast Thailand (Fig. 3).

### *Ov*-TSP-2 and *Ov*-TSP-3 are expressed in the tegument and eggs of *O. viverrini* adult worms

**Figure 4:**
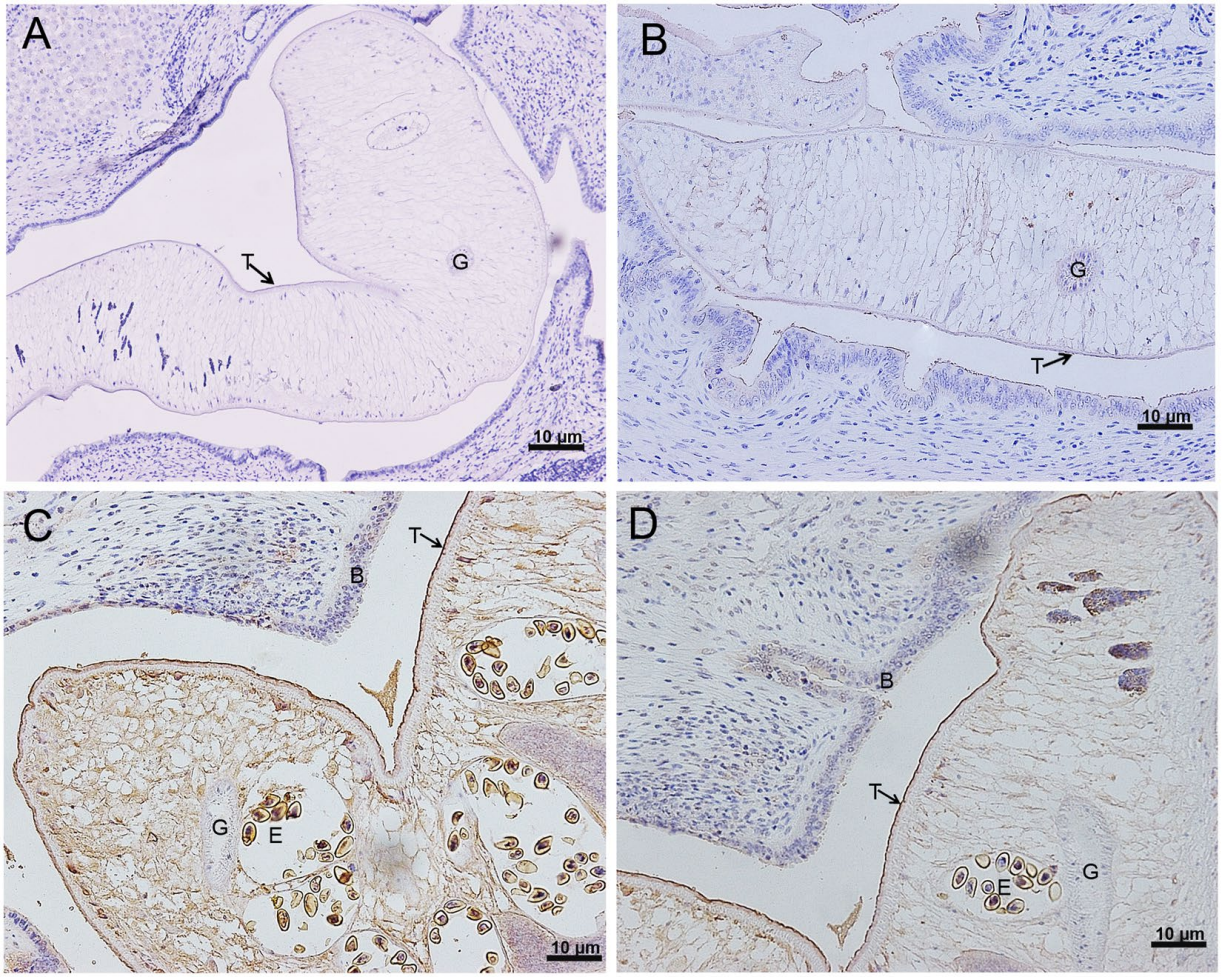
Immunohistochemical detection of *Ov*-TSP-2 and *Ov*-TSP-3 in *Opisthorchis viverrini* from the bile ducts of infected hamsters. Panels A and B: Liver sections of infected hamsters probed with pre-immunization sera showing no immunoreactivity. Panels C and D: Liver sections of infected hamsters probed with rabbit anti *Ov*-TSP-2 (**C**) and *Ov*-TSP-3 (**D**) sera showed positive staining in the tegument (T) and eggs (E) within the uteri of the flukes and in the biliary epithelium (B) of hamsters adjacent to the flukes but not in the gastrodermis (G) of the parasite.

Sections from the bile ducts of infected hamsters were probed with sera against *Ov*-TSP-2 or TSP-3, followed by secondary anti-rabbit antibodies conjugated to horseradish peroxidase (HRP) and developed with 3,3′ Diaminobenzidine (DAB). Both *Ov*-TSP-2 and TSP-3 were strongly detected on the tegument surface and eggs of *O. viverrini*. Interestingly, positive staining was also observed in the hamster biliary epithelium adjacent to the fluke when probed with rabbit anti *Ov*-TSP-2 and *Ov*-TSP-3 (Fig. 4).

### *Ov*-TSP-2 and *Ov*-TSP-3 are expressed throughout different developmental stages of *O. viverrini*

**Figure 5:**
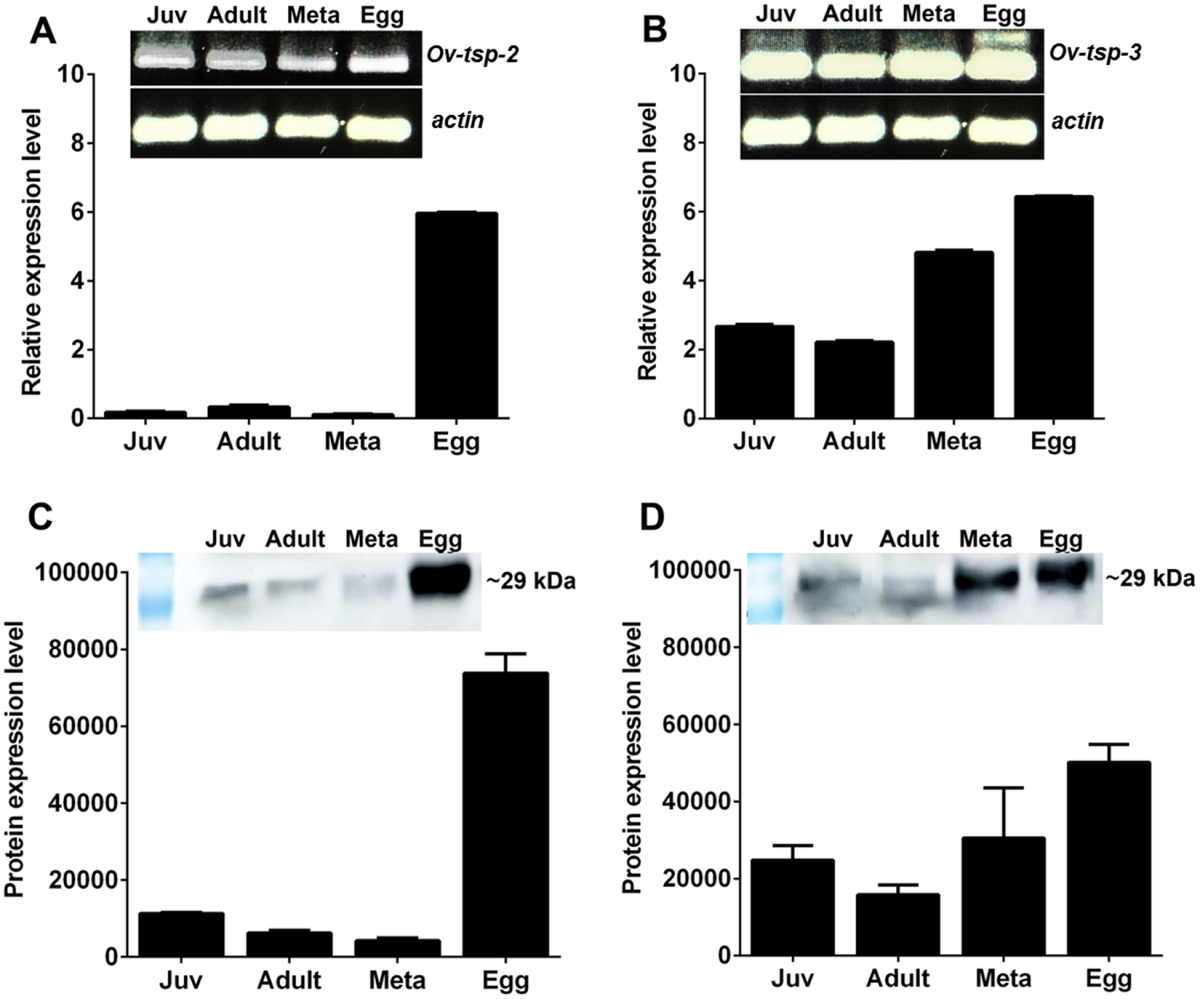
Expression levels of *Ov-tsp-2* and *Ov-tsp*-3 in different developmental stages of *Opisthorchis viverrini*. Expression levels of *Ov-tsp-2* (**A**) and *Ov-tsp-3* (**B**) mRNAs relative to *Ov-actin* were analysed by qRT-PCR. Protein expression levels in the same developmental stages were evaluated by Western blot probed with rabbit anti-*Ov*-TSP-2 (**C**) or rabbit anti-*Ov*-TSP-3 (**D**); Western blot band intensity was quantified using densitometry. The experiments were performed in biological duplicate and represented as the mean ± SD. Juvenile (Juv), *O. viverrini* adult worm (Adult), Metacercariae (Meta). Supplementary Fig. S3 provides the full length ethidium bromide stained agarose gels of RT-PCR products, and Supplementary Fig. S4 provides the full length western blot membrane strips.

The transcriptional patterns of both *Ov*-*tsp-2* and *Ov*-*tsp-3* were analysed in different developmental stages of *O. viverrini* (i.e. adult fluke, two-week-old juvenile fluke, metacercariae and eggs) by qRT-PCR. Both mRNAs were expressed through all assessed stages of development. Expression levels were compared to the actin gene (*Ov-actin*). *Ov-tsp-2* expression peaked in eggs, then declined in subsequent developmental stages by 55-, 35- and 19-fold in metacercariae, juvenile flukes and adult worms, respectively (Fig. 5A; P < 0.001). Similarly, expression of *Ov-tsp-3* was highest in eggs, decreased at least 2-fold in the subsequent developmental stages (Fig. 5B; P < 0.001). Likewise, the *Ov*-TSP-2 and TSP-3 protein levels were greatest in eggs, particularly for TSP-2 (Fig. 5C and D), and fell more than 10-fold in other developmental stages stages (Fig. 5C; P < 0.001). The protein levels of *Ov*-TSP-3 were also highest in eggs, and decreased 2- and 3-fold in adult (P = 0.0018) and juvenile flukes (P = 0.0019), respectively (Fig. 5D).

### dsRNA-mediated knockdown of *Ov-tsp*-2 and *Ov-tsp*-3 expression

**Figure 6:**
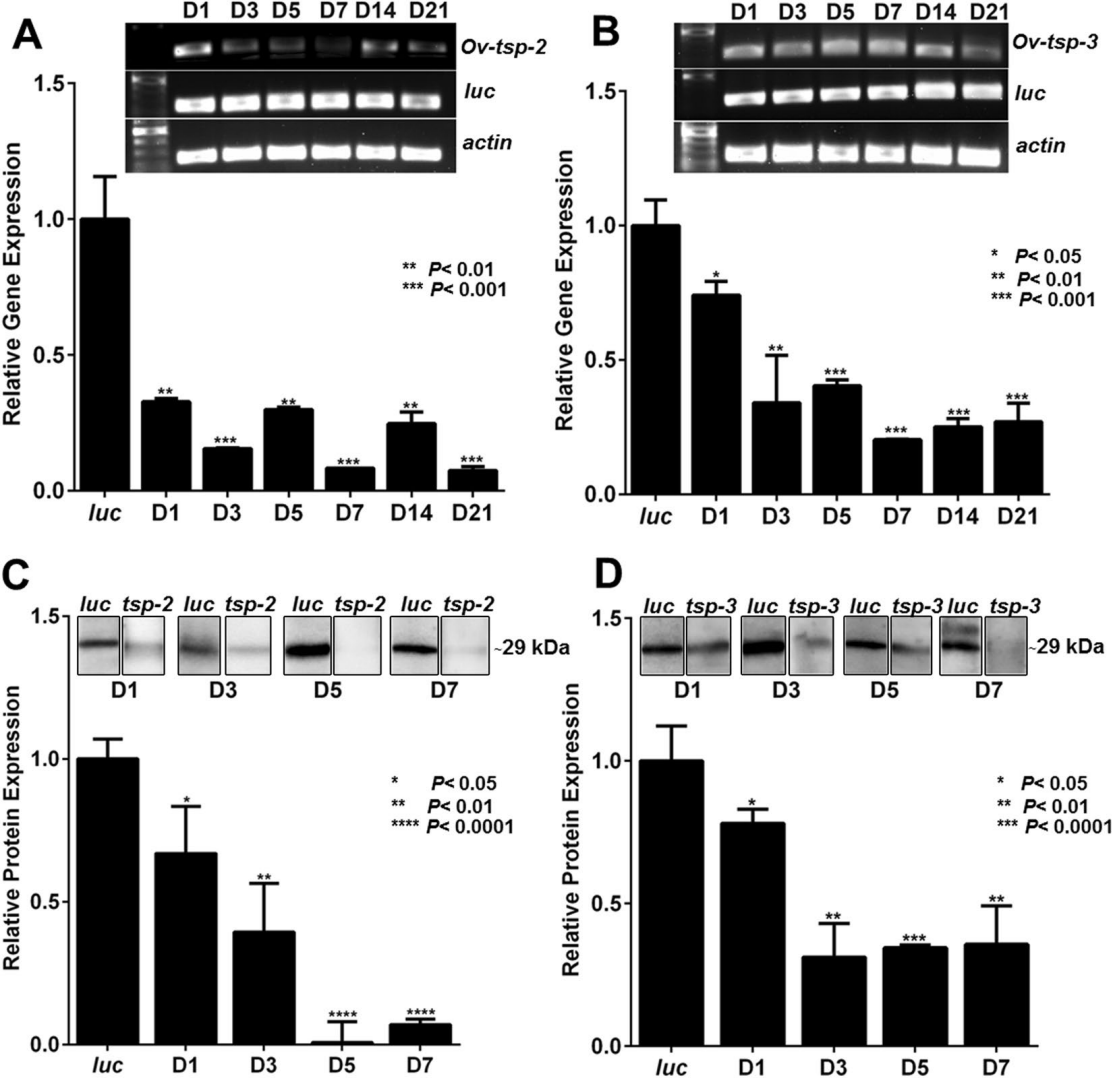
Suppression of *Ov-tsp-2* and *Ov-tsp-3* mRNA expression in adult *O. viverrini* flukes by RNA interference. mRNA expression levels of *Ov-tsp-2* (**A**) and *Ov-tsp-*3 (**B**) relative to *Ov-actin* (mean ± S.E.) in adult worms electroporated with 50 µg dsRNA of *Ov-tsp-2* or *Ov-tsp-*3 or *luciferase* control followed by soaking in culture media containing 2 µg/ml dsRNA for 21 days. mRNA expression was measured by qRT-PCR. The protein expression levels of *Ov*-TSP-2 (**C**) and *Ov*-TSP-3 (**D**) in worms after electroporation with dsRNAs were analysed by Western blot and quantified using imageJ. The experiments were performed in biological duplicate and represented as the mean ± SD. Supplementary Fig. S5 provides the full length ethidium bromide stained agarose gels of RT-PCR products, and Supplementary Fig. S6 provides the full length western blot membrane strips.

dsRNAs corresponding to *Ov-tsp-2* and *tsp-3* were introduced into adult worms by square wave electroporation and soaking of flukes for 1, 3, 5, 7, 14 and 21 days. The survival rates of *Ov-tsp-2* and Ov-*tsp-3* knocked down worms were 88% and 94% for 21 days, respectively; no differences were detected compared to negative control groups that received *luc* dsRNA (not shown). An 80% reduction of mRNA expression was detected by day 3 in adult worms treated with *Ov-tsp-2* dsRNA. Expression levels of *Ov-tsp-2* were reduced by 84%, 70%, 92%, 75% and 98% on days 3, 5, 7, 14, 21, respectively after exposure to *tsp-2* dsRNA (Fig. 6A). Similarly, the expression levels of *Ov-tsp-3* declined steadily to 80% reduction on day 7 before returning to 70% and 73% reductions on days 14 and 21, respectively (Fig. 6B).

To determine whether knockdown of *Ov-tsp-2* and *Ov-tsp-3* mRNAs was evident at the protein level, adult worms were treated with *Ov-tsp-2*, or *Ov-tsp-3*, or *luciferase* as control for 7 days. *O. viverrini* whole worm extract was immunoblotted with antibodies raised to *Ov*-TSP-2 and TSP-3. The amount of *Ov*-TSP-2 protein expression was reduced by more than 90% after knockdown of *Ov-tsp-2* at day 5 (Fig. 6C). Likewise *Ov-*TSP-3 protein expression was suppressed by 85% after *tsp-3* dsRNA-treatment at day 7 (Fig. 6D) when compared to the irrelevant dsRNA electroporated control group.

### Suppression of *Ov-tsp-2* and *Ov-tsp-3* mRNAs results in malformation of the tegument

**Figure 7:**
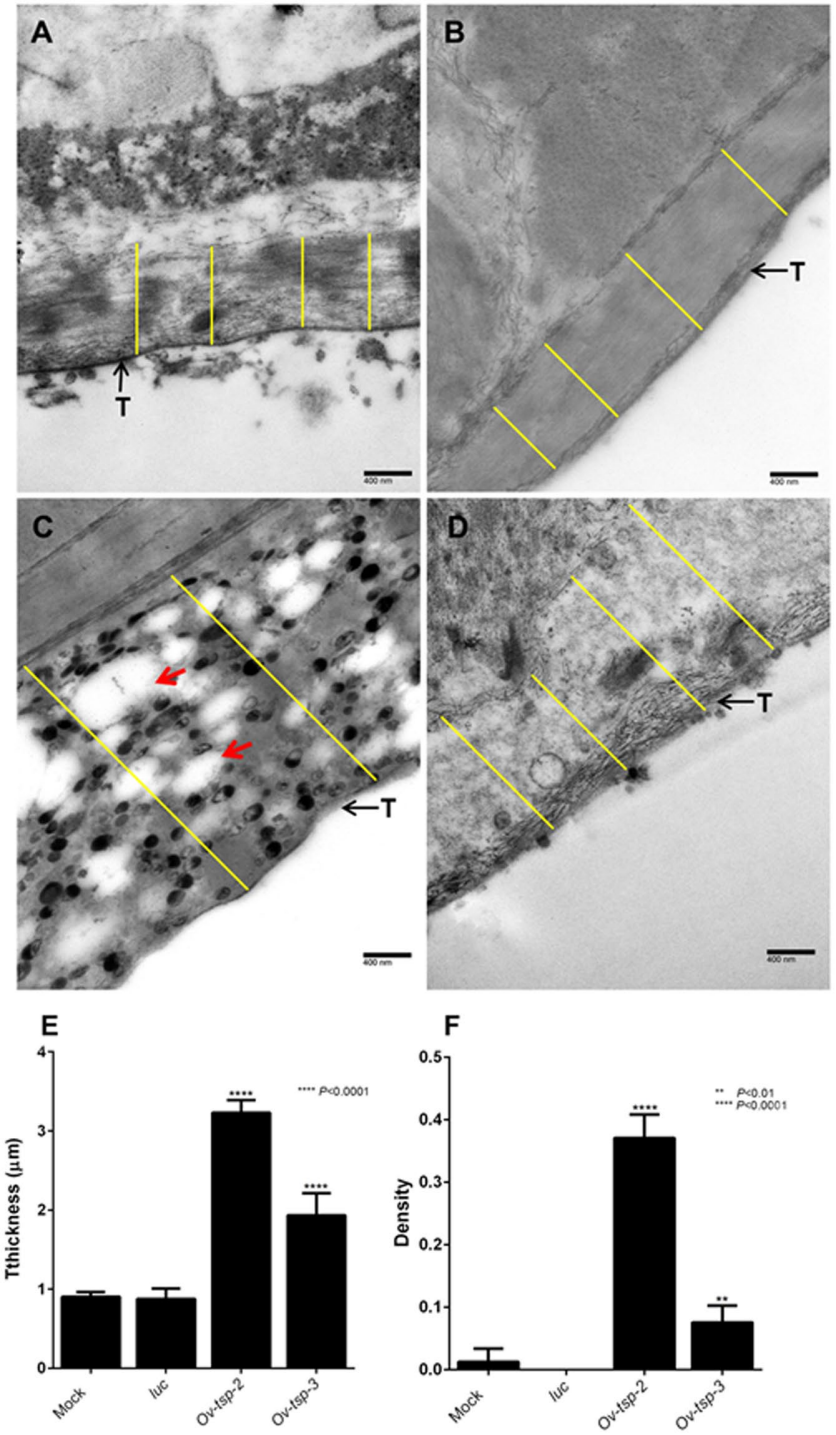
Ultrastructure of the tegument of *Opisthorchis viverrini* adult worms treated with *Ov-tsp-2* and *tsp-3* dsRNAs. *O. viverrini* adult worms were electroporated with RPMI medium (**A**) or the mock control firefly *luciferase* dsRNA (**B**), *Ov-tsp-2* dsRNA (**C**), and *Ov-tsp-3* dsRNA (**D**), further cultured in RPMI medium containing 2 µg dsRNA for 3 days, and analysed using transmission electron microscopy (magnification 40,000x). The tegument (T) of worms treated with *Ov-tsp-2* dsRNA was thicker than controls (P < 0.0001) (Fig. 7C,E) and displayed extensive vacuolation (P < 0.0001) (Fig. 7C,F; red arrows).The tegument of *Ov-tsp-3* dsRNA-treated worms was also thicker (P < 0.0001) (Fig. 7D,E) and less electron dense when compared to controls (Fig. 7D,F). The lines indicate the width of fluke tegument that was used for measurement of tegument thickness.

To investigate the effects of silencing the expression of tetraspanin mRNAs, *O. viverrini* adult worms treated with dsRNAs targeting *Ov-tsp-2* or *Ov-tsp-3* and subsequently cultured for 3 days. Using transmission electron microscopy (TEM) we showed that the tegument of *O. viverrini* exposed to *Ov-tsp-2* dsRNA (Fig. 7C) was 4-fold thicker than the tegument of flukes treated with irrelevant dsRNAs (P < 0.0001; Fig. 7E), measuring on average 3.2306 ± 0.040 µm compared with 0.8770 ± 0.033 µm for *luciferase* controls (Fig. 7B). Likewise, the tegument of *Ov-tsp2* dsRNA-treated worms was more densely vacuolated (0.370 vacuolar density score; P < 0.0001; Fig. 7C,F) when compared with *luc* control flukes (0.000 vacuolar score; Fig. 7B,F). The tegument of worms exposed to *Ov-tsp-3* dsRNA was significantly thicker on average (1.933 ± 0.007 µm; P < 0.0001; Fig. 7D,E), and less electron dense (0.075 vacuolar score; P = 0.0083; Fig. 7D,F) compared to the tegument of *luc* dsRNA-treated flukes.

## Discussion

Tetraspanins are a ubiquitously distributed family of proteins containing four transmembrane domains and two extracellular loops - the SEL and LEL^15^. Tetraspanins are involved in numerous functions, including cell adhesion, motility, fusion, signaling and also host-parasite interactions^16^, and play important roles in protein-protein interactions and in the molecular organization of cell membranes^17^. We previously characterized a member of the CD9/81 tetraspanin lineage from *O. viverrini*, *Ov*-TSP-1, which is expressed on the tegument of adult worms^8^. Herein, two tetraspanins from the CD63 lineage of *O. viverrini* were characterized and their role in tegument architecture was analyzed using gene silencing approaches.

*Ov*-TSP-2 and TSP-3 contain many of the conserved features found in the CD63 family of TSPs. *Sm*-TSP-2 is a CD63-like TSP found in the tegument of the parasitic blood fluke, *Schistosoma mansoni*. Silencing the expression of the *Sm-tsp-2* gene resulted in malformed tegument of *S. mansoni* and proved lethal when dsRNA-treated larval parasites were transferred into mice^7^. *Sm*-TSP-2 LEL is an efficacious vaccine in mouse models of schistosomiasis^11^ and is recognised by sera from naturally resistant human subjects in a disease endemic area of Brazil^18^. Using cross-linking agents, *Sm*-TSP-2 was shown to bind to at least 8 other tegument proteins, some of which had already been shown to have vaccine efficacy in mice or non-human primates, including calpain, HSP70, actin and annexin^19^. The tegument proteome of *O. viverrini* contains homologues of most of these proteins^9^, but whether they interact with *Ov*-TSP-2 or TSP-3, or indeed whether these two TSPs interact with each other, remains to be determined.

*Ov-tsp-2* and *Ov-tsp-3* mRNAs were expressed throughout the life cycle of the parasite with the highest expression in eggs. Moreover, both proteins were detected by immunohistochemistry in the egg shell and the developing larva contained within the egg, as well as the adult fluke tegument. These findings, implying that *Ov*-TSP-2 and *Ov*-TSP-3 might play important roles in the formation and/or stabilization of the cellular surface throughout the development of the parasite, from the egg shell surface right through the developmental phases of parasite maturation in the molluscan, fish and definitive mammalian hosts. In contrast, *Ov*-TSP-1 was highly expressed in metacercariae, and detected immunologically at the tegument surface of adult flukes^8^.

Interestingly, *Ov*-TSP-2 and TSP-3 were detected on or inside the biliary epithelial cells of infected hamsters in the vicinity of resident flukes. *Ov*-TSP-2 and TSP-3 are located within the tegument^9^ but other TSPs have also been identified in extracellular vesicles (EVs) secreted by *O. viverrini* ^10^. Given that *O. viverrini* EVs are internalized by cholangiocytes *in vitro* ^20^, it is not altogether surprising that we detected *Ov*-TSP-2 and TSP-3 on (or in) the cholangiocytes lining the bile ducts of infected hamsters, although the presence of these two tetraspanins in EVs has yet to be determined definitively. Indeed, internalization of *O. viverrini* EVs by cholangiocytes resulted in cell proliferation and production of inflammatory cytokines, thereby promoting a tumorigenic environment^10^. Tetraspanins are highly abundant on the surface membranes of EVs released by mammalian cells and are considered as diagnostic markers of exosomes^21,22^. Tetraspanins might contribute to exosome assembly and could be important for target cell selection and the tight interactions and cell membrane fusions that have been reported during exosome uptake by target cells^21^, however the specific mechanisms of exosome internalization remain unclear.

The important role of tetraspanins in the molecular organization of *Opisthorchis* cell membranes was demonstrated using RNA interference. Silencing of *Ov-tsp-2* and *Ov-tsp-*3 mRNA expression resulted in impaired tegument formation in adult flukes. When *Ov-tsp-2* was silenced, large vacuoles developed in the tegument cytoplasm. When *Ov-tsp-3* was silenced, an effect on vacuolation was less apparent but instead the tegument of these flukes was thinner than that of control flukes treated with *luc* dsRNA. In *S. mansoni*, silencing of *Sm-tsp-2* expression in schistosomula larvae and adult worms also resulted in a vacuolated and thinner tegument^7^. RNAi has also been used to determine the importance of tetraspanins in free-living helminths such as the nematode *Caenorhabditis elegans*, in which suppression of *tetraspanin-15* mRNA expression resulted in dissociation of the cuticle and degeneration of the hypodermis, compromising epidermal integrity^23^.

We have characterized for the first time two members of the CD63 lineage of tetraspanins in the carcinogenic liver fluke, *O. viverrini*. Our findings show that *Ov*-TSP-2 and TSP-3 are essential for tegument membrane formation in liver flukes, and therefore present as potential antigens for inclusion in a subunit vaccine against liver fluke infection.

## Materials and Methods

### Experimental animals and *O. viverrini*

*O. viverrini* metacercariae were collected from naturally infected cyprinoid fish as previously described^24^. Fifty metacercariae were used to infect 6–8 week-old Golden Syrian hamsters (*Mesocricetus auratus*) by the oral route using intragastic tube feeding. Hamsters were euthanized, blood was obtained by cardiac puncture and worms were recovered from the gall bladders and bile ducts. Juvenile and adult *O. viverrini* worms were harvested from infected hamster livers at 2 weeks and 4 weeks, respectively. Eggs of *O. viverrini* were obtained from cultured worms in RPMI supplemented with 1x antibiotics (Penicillin-Streptomycin 100 µg, Invitrogen, USA) and centrifuged at 2,090 *g* for 10 min. Hamsters were reared at the animal facility, Faculty of Medicine, Khon Kaen University. New Zealand white rabbits were purchased from the National Laboratory Animal Center, Mahidol University, Thailand. Rabbits were maintained in the Northeast Laboratory Animal Center, Khon Kaen University, and were vaccinated with recombinant proteins as described below. All experimental procedures performed in this study were conducted in accordance with, and by approval of the Animal Ethics Committee of Khon Kaen University according to the Ethics of Animal Experimentation of the National Research Council of Thailand, approval number ACUCKKU10/2559.

### Preparation of *O. viverrini* excretory-secretory products

*O. viverrini* excretory-secretory products (*Ov*ES) were prepared as previously described^25,26^. Briefly, adult worms were maintained in RPMI-1640 containing 1% glucose, 1 µM E64 protease inhibitor (Thermo Scientific, USA) and antibiotic (Penicillin-Streptomycin 100 µg, Invitrogen, USA). Worms were cultured at 37 °C with 5% CO_2_, culture medium was harvested twice a day for 7 days. Culture medium was pooled and centrifuged at 2,090 *g* for 10 min to remove eggs and concentrated using an Amicon 10 kDa cut-off centrifugal filter (Merck, USA).

### Cloning of *O. viverrini* cDNAs encoding *Ov-tsp-2* and *tsp-3*

The cDNAs encoding for the open reading frames (ORFs) of *Ov*-*tsp-2 and tsp-3* were obtained by PCR from an adult worm cDNA library^24^. Oligonucleotide primers for PCR to amplify the complete ORFs were designed based on expressed sequence tags (ESTs)^24,27^. The primers used for *Ov-tsp-2* were Ov-TSP2F (5′-AGTAATGGTCTCGCTAAGTTGTGG-3′) and Ov-TSP2R (5′-ACTGTCTATACCGTCTCGCCTTCTCC-3′). The primers used for *Ov-tsp-3* were Ov-TSP3F (5′-ACGAATATGGTCTCCCTCAGCTGTGGC-3′) and Ov-TSP3R (5′-ACCATTTATGCATCTTCACCGCGTTG -3′); positions of start and stop codons are underlined. PCR products were sequenced before ligation into the pGEM T Easy vector (Promega, USA), and sequences determined using the BigDye terminator method (1^st^ BASE, Singapore).

### Sequence analysis and Phylogenetic analysis

DNA sequences were evaluated using BioEdit V7.2.5^28^. The edited sequences were translated to protein using web based translation software at http://bio.lundberg.gu.se/edu/translat.html and compared to related sequences using the basic local alignment search tool (BLAST) at http://blast.ncbi.nlm.nih.gov/ ^29^ Signal peptides from the deduced amino acid sequences were predicted by SignalP 3.0 (http://www.cbs.dtu.dk/services/SignalP-3.0/). Other divergent sequences were compared via multiple alignment using ClustalW in the BioEdit program. Transmembrane regions were predicted using the TMpred Server (www.ch.emnet.org/software/TMPRED_form.html) and two-dimensional structures were designed by Protter Sever (http://wlab.ethz.ch/protter/start/).

Phylogenetic relationships among TSPs from a range of organisms were constructed based on amino acid sequences. ORFs were aligned using ClustalW^30^. A phylogenetic tree was constructed with p-distance matrix using the neighbor-joining method^31^ with 1,000 bootstrap samplings in the MEGA software package version 6.0.6^32^.

### Production of recombinant *Ov*-TSP-2 and TSP-3 large extracellular loop regions

The large extracellular loop (LEL) regions of *Ov-*TSP-2 (amino acid residues 109–185) and *Ov-*TSP-3 (amino acid residues 110–184) were obtained by PCR using plasmid containing the full length mRNA sequences of *Ov-tsp-2* and *tsp-3* (as described above) as templates. The primers for LEL of *Ov*-TSP-2 were: forward primer TSP2_ECF 5′-ACGCGAATTCCGCGATAAGATCCCCGG-3′, and reverse primer TSP2_ECR 5′-ACGCGCGGCCGCCTGGATGAACTCTTCGAC-3′; primers for LEL of Ov-TSP-3 were: forward primer; TSP3_ECF 5′ACGCGAATTCGACCATGTGAAAGAA-3′, and reverse primer; TSP3_ECR, 5′ACGCGCGGCCGCTTCGATGAATTTATC-3′, respectively. The fragments were integrated into the *Eco*RI and *Not*I sites (underlined) to facilitate cloning into the vector in frame with the N-terminal TRX and 6x His tags of pET-32a (Novagen, USA). PCR conditions were 35 cycles of denaturation at 95 °C for 30 sec, annealing at 55 °C for 30 sec, extension at 72 °C for 1 sec, and final extension at 72 °C for 10 minutes. PCR products were subsequently digested with *Eco*RI and *Not*I, and fragments cloned into pET-32a (Novagen, USA). Recombinant plasmids were designated as *pET32a-LEL-Ov-TSP-2* or *pET32a LEL-Ov-TSP-3*. The insert identity and in-frame fusion to the 6x His tag were confirmed by sequencing using the BigDye terminator method (1^st^ BASE, Singapore). *pET32a-LEL-Ov-TSP-2* and *pET32a-LEL-Ov-TSP-3* were transformed into BL21DE3 strain *E. coli* (Novagen, USA). The recombinant clones were screened for ampicillin resistance, and PCR amplified using T7 and reverse specific primers, either Ov-TSP2_ECR or Ov-TSP3_ECR for confirmation of insertion of sequences. The transformed bacteria were induced with 1 mM IPTG in LB broth for 4–6 hours at 37 °C with horizontal shaking to produce recombinant proteins. The proteins were purified by Ni-NTA affinity chromatography according to the manufacturer’s instruction (His-Bind Resin, Novagen) under non-denaturing conditions with 500 mM imidazole, dialyzed into PBS and analyzed by Coomassie-stained SDS-PAGE.

### Synthesis of double-stranded RNAs (dsRNAs)

Double-stranded RNAs (dsRNAs) of *Ov-tsp-2*, *Ov-tsp-3* and firefly luciferase (*luc*) were constructed using a MEGA script RNAi kit (Amicon), following the manufacturer’s instructions. *Ov-tsp-2* and *Ov-tsp-3* dsRNAs were synthesized from plasmids using primers flanked with a T7 RNA polymerase promoter sequence at the 5′ end. The *Ov-tsp-2* dsRNA of 556 bp was generated using primers ds-TSP2_T7-F (5′***-TAATACGACTCACTATAGGG***GGCCTCATAGTTGTCGGGAGT3′) and ds-TSP2_T7-R (5′-***TAATACGACTCACTATAGGG***GCACAACACAGATGGCAA3′), and a 529 bp fragment of the *Ov-tsp3* dsRNA using primer ds-TSP3_T7-F (5-***TAATACGACTCACTATAGGG***AGCATCGCCCAAGTCCAGCTG3′) and ds-TSP3_T7-R (5′-***TAATACGACTCACTATAGGG***GCGTTGAATCGCCTTGCA CAC3′). *luc* dsRNA was derived using primer ds-LUC_T7-F (5′-***TAATACGACTCACTATAGGG***TGCG CCCGCGAACGACATTTA3′) and ds-LUC_T7-F (5′-***TAATACGACTCACTATAGGG***GCAACCGCTTCCCCGACTTCCTTA3′). The PCR products were amplified using 35 cycles of denaturation at 95 °C for 30 sec, annealing at 55 °C for 30 sec, extension at 72 °C for 1 sec, and final extension at 72 °C for 7 minutes.

### Delivery of dsRNA by electroporation

Adult worms were washed with PBS three times prior to electroporation. For dsRNA electroporation, 21 worms recovered from hamster bile ducts in each group were placed in 100 µl of culture media containing 50 µg *Ov-tsp2*, *Ov-tsp3*, or *luc* dsRNA in a 4-mm gap electroporation cuvette (Bio-Rad, Hercules, CA, USA). Samples were single pulsed by square wave electroporation at 125 V, 20 ms duration (Electroporation Gene Pulser Xcell, Bio-Rad). After pulsing, worms were soaking with 2 µg dsRNA in 1% glucose RPMI at 37 °C with 5% CO_2_ atmosphere for 21 days with changes of media and dsRNAs every second day. Parasites were harvested at days 1, 3, 5, 7, 14 and 21 and washed prior to RNA or protein extraction. For transmission electron microscopy, dsRNA-treated worms were collected on days 3 and 5, and fixed in 3% glutaraldehyde in 0.1 M phosphate buffer at pH 7.4. In terms of survival and mortality rates after gene silencing, thirty adult worms were electroporated (single pulse) in media containing 50 µg *Ov-tsp2*, *Ov-tsp3*, or *luc* dsRNAs in a 4-mm gap electroporation cuvette. After electroporation, the worms were maintained in RPMI supplemented with 1% glucose, E64 cysteine protease inhibitor and antibiotic containing 2 µg dsRNA for 21 days. Electroporated worms were visually monitored for viability on a daily basis by observing fluke movement and sucker movement using light microscopy. Worms were scored for viability if oral or ventral suckers were still moving (alive); if no movement was apparent during a 10 min observation period the worms were considered to be dead^33^. The experiment was done in duplicate. Statistical analyses were performed with a Student’s *t*-test student for comparison between groups using GraphPad Prism Software Version 6.03 (www.graphpad.com).

### RNA preparation and RT-PCR

Total RNA from *O. viverrini* adults, juvenile flukes, metacercariae and eggs were extracted with TRIZOL (Invitrogen, USA) supplemented with RNase-free DNase I (Invitrogen). For RT-PCR, 1.0 µg of total RNA was reverse-transcribed to cDNA using a RevertAid First Strand cDNA synthesis kit (thermo scientific, USA), and qRT-PCR was performed by a LightCycler 480 (Roach Diagnostics, Germany) with SYBR green I as the detection fluorophore and using the following primers: *Ov*-tsp-2 primer: forward: (5′-GGTCTCGCTAAGTTGTGG-3′) and reverse (5′-GCATGCTCCACAGAACCCC-3′); *Ov*-tsp-3 primer: forward: (5′GCTGTGGCTA CAAGTGTTTGC-3′) and reverse (5′-CCAAAGCTTCCGACAACAGT-3′), which generated fragment sizes at 229 and 202 bp in length, respectively. Real-time qRT-PCR was performed in triplicate. SYBR qRT-PCR reaction consists of 10 µl of SYBR premix EX Taq (2x), 0.4 µl (10 mM) of forward and reverse primer, 2 µl (100 ng) of the first–stand cDNA and sterile water to final volume 20 µl. The PCR cycling condition was followed by initiation pre-heat at 95 °C for 1 cycle, 40 cycles of PCR step which is denaturation at 95 °C for 5 sec, annealing at 60 °C for 30 sec and extension at 72 °C for 1 sec. The mRNA expression of candidate genes were normalized with actin mRNA (OvAE1657, GenBank EL620339.1) as internal control. dsRNA- treated worms were collected at specific time points, and washed extensively in PBS. Total RNA was extracted using TRIZOL and cDNA synthesis was performed as described above. *Ov-tsp-2* and *Ov-tsp-3* transcript levels were normalized to *Ov-actin* mRNA levels and irrelevant dsRNA-treated parasites, and presented using the 2^−ΔΔCt^ method where ΔΔCt = ΔCt (treated worms) − ΔCt (non-treated worms)^34^. All experiments were performed in duplicate.

### Production of polyclonal anti-*Ov*-TSP2 and anti-*Ov*-TSP-3

New Zealand white rabbits were immunized 4 times with 500 µg adjuvanted recombinant proteins at two weekly intervals. Blood was collected from ear veins of each rabbit before immunization and by cardiac puncture two weeks after the last immunization at euthanasia.

### Western blotting

Whole worm extract from adult worms, eggs and metacercariae were extracted in Tris buffer (50 mM Tris-HCl, 150 mM NaCl, 1% Triton-X100), homogenized by sonication (5 sec interval) for 45 sec and centrifuged at 12,000 *g* for 10 min to remove cell debris. *Ov-tsp-2* and *Ov-tsp-3* dsRNA-treated adult worms were cultured as described above and harvested on days 1, 3, 5, and 7, following extraction in Tris buffer. Protein concentrations were determined by Nanodrop 2000 spectrophotometer (Thermoscientifiic, USA) and 3 µg of lysates were electrophoresed in 15% SDS-PAGE gels. Proteins were transferred to nitrocellulose membrane (Mini Trans-Blot Cell, Bio-Rad), with PBST (1x PBS + 0.01% Tween-20), blocking was achieved using 5% skim milk and probed with either rabbit anti-*Ov*-TSP-2 or rabbit anti-*Ov*-TSP-3 at a dilution 1:1,000 overnight at 4 °C followed by anti-rabbit IgG-HRP (Merck millipore, USA) dilution 1:1,000. To identify antibodies in infected humans and hamsters, recombinant *Ov*-TSP-2 and *Ov*-TSP-3 were transblotted, probed with parasite-infected human or hamster serum (1:500) followed by secondary antibodies conjugated to HRP (1:1,000). The bands were visualized with ECL chemiluminescence detection (Luminata Forte Western HRP substrate, Merck Millipores, USA) and photo with Luminescent Image analysis (ImageQuant LAS 4000mini, GE healthcare, USA). The quantification of Western blot data was achieved using ImageJ software (https://imagej.nih.gov/ij/). Sera of naturally infected human subjects from an endemic area in northeast Thailand were provided by Dr. Banchob Sripa, Tropical Disease Research Laboratory, Department of Pathology, Faculty of Medicine, Khon Kaen University with approval from the Khon Kaen University Ethics committee for human research (approval number HE480528).

### Immunohistochemistry

Liver sections from *O. viverrini*-infected hamsters embedded in paraffin were de-parafinized using xylene and rehydrated in an ethanol series (100%, 90%, 80% and 70% ethanol for 5 min each). Sections were immersed in citrate buffer (pH 6), boiled in a pressure pot for 5 min and subsequently blocked using 3% H_2_O_2_ in methanol. They were incubated with rabbit anti-*Ov*-TSP-2, or anti-*Ov*-TSP-3 sera diluted 1:200 in PBS, overnight at 4 °C. Sections were finally probed with goat anti-rabbit IgG-HRP (Invitrogen, USA) diluted 1:1000 in PBS. Peroxidase reaction products were visualized with 3,3′-diaminobenzidine (DAB) (Sigma-Aldrich). Counterstaining was performed by Mayer’s hematoxylin for 5 min. A positive signal was indicated by a reddish-brown color under light microscopy.

### Transmission electron microscopy (TEM)

Electroporated worms treated with dsRNAs of *Ov-tsp-2*, *Ov-tsp-3* or *luc* as described above were subjected to transmission electron microscopy (TEM) analysis to examine for potential tegument impairment. Briefly, worms were washed three times in 1x PBS and then fixed in 2% glutaraldehyde in 0.1 M phosphate, pH 7.4 for 48 h followed by fixation in 1% osmium tetroxide in phosphate buffer. After washing with phosphate buffer, they were dehydrated in 50%, 70%, 80%, 90%, 95% and absolute ethanol. Finally, worms were embedded in epon 812 (Electron Microscopy Sciences, Hatfield, PA) and polymerized at 60 °C for 48 hours. Ultrathin sections mounted onto copper grids were observed under a JEOL transmission electron microscope (JEM1010, Tokyo, Japan, equipped with a digital camera) at 100 kV.

The volume density of vacuolar compartments and thickness of tegument were analysed by Image J analysis software (NIH, Bethesda) based on the method described by Tran^7^ with minor modifications. Briefly, three TEM images (x20,000 magnification) were used to estimate the volume density of vacuolar compartments of the tegument of *O. viverrini*. A 0.4 µm^2^ grid was drawn on the image. The density of vacuoles was estimated by determining the number of vacuoles in the grid intersect divided by the number of grid intersects of the examined tegument area. The thickness of the tegument was measured using the line tool in Image J software. Each image was measured at 15 different points. The line tool was calibrated before measuring and drawn digitally on each point from the basal membrane to the apical membrane of tegument. Mean and standard deviations were calculated for each group.

### Statistical analyses

Data was expressed as the mean ± standard error. Statistical *t*-test analyses were performed using GraphPad Prism Software Version 6.03 (www.graphpad.com). Statistically significant differences for a particular comparison were defined as p < 0.05.

### Gene accession number

Sequences of *O. viverrini* tetraspanins, *Ov-tsp-2* and *Ov-tsp-3* were submitted to GenBank database and were assigned the accession numbers JQ678707 and JQ678708, respectively.

## Electronic supplementary material

Supplementary figures

## References

[1] Sithithaworn P (2012). The current status of opisthorchiasis and clonorchiasis in the Mekong Basin. Parasitology international.

[2] IARC. Biological agents. Volume 100 B (2012). A review of human carcinogens. IARC monographs on the evaluation of carcinogenic risks to humans / World Health Organization, International Agency for Research on Cancer.

[3] Sripa B (2012). The tumorigenic liver fluke Opisthorchis viverrini–multiple pathways to cancer. Trends Parasitol.

[4] Smout MJ (2009). A granulin-like growth factor secreted by the carcinogenic liver fluke, Opisthorchis viverrini, promotes proliferation of host cells. PLoS pathogens.

[5] Young ND (2011). A portrait of the transcriptome of the neglected trematode, Fasciola gigantica–biological and biotechnological implications. PLoS neglected tropical diseases.

[6] Levy S, Shoham T (2005). The tetraspanin web modulates immune-signalling complexes. Nat Rev Immunol.

[7] Tran MH (2010). Suppression of mRNAs encoding tegument tetraspanins from Schistosoma mansoni results in impaired tegument turnover. PLoS pathogens.

[8] Piratae S (2012). Molecular characterization of a tetraspanin from the human liver fluke, Opisthorchis viverrini. PLoS neglected tropical diseases.

[9] Mulvenna J (2010). The secreted and surface proteomes of the adult stage of the carcinogenic human liver fluke Opisthorchis viverrini. Proteomics.

[10] Chaiyadet S (2015). Carcinogenic Liver Fluke Secretes Extracellular Vesicles That Promote Cholangiocytes to Adopt a Tumorigenic Phenotype. The Journal of infectious diseases.

[11] Tran MH (2006). Tetraspanins on the surface of Schistosoma mansoni are protective antigens against schistosomiasis. Nat Med.

[12] Pinheiro CS (2014). A multivalent chimeric vaccine composed of Schistosoma mansoni SmTSP-2 and Sm29 was able to induce protection against infection in mice. Parasite Immunol.

[13] Goncalves de Assis NR (2015). DNA Vaccine Encoding the Chimeric Form of Schistosoma mansoni Sm-TSP2 and Sm29 Confers Partial Protection against Challenge Infection. PloS one.

[14] Garcia-Espana A (2008). Appearance of new tetraspanin genes during vertebrate evolution. Genomics.

[15] Maecker HT, Todd SC, Levy S (1997). The tetraspanin superfamily: molecular facilitators. FASEB J.

[16] Stipp CS, Kolesnikova TV, Hemler ME (2003). Functional domains in tetraspanin proteins. Trends Biochem Sci.

[17] Hemler ME (2005). Tetraspanin functions and associated microdomains. Nat Rev Mol Cell Biol.

[18] Gaze S (2014). An immunomics approach to schistosome antigen discovery: antibody signatures of naturally resistant and chronically infected individuals from endemic areas. PLoS pathogens.

[19] Jia X (2014). Solution structure, membrane interactions, and protein binding partners of the tetraspanin Sm-TSP-2, a vaccine antigen from the human blood fluke Schistosoma mansoni. The Journal of biological chemistry.

[20] Chaiyadet S (2015). Excretory/secretory products of the carcinogenic liver fluke are endocytosed by human cholangiocytes and drive cell proliferation and IL6 production. International journal for parasitology.

[21] Rana S, Zoller M (2011). Exosome target cell selection and the importance of exosomal tetraspanins: a hypothesis. Biochem Soc Trans.

[22] Zoller M (2009). Tetraspanins: push and pull in suppressing and promoting metastasis. Nat Rev Cancer.

[23] Moribe H (2004). Tetraspanin protein (TSP-15) is required for epidermal integrity in Caenorhabditis elegans. J Cell Sci.

[24] Laha T (2007). Gene discovery for the carcinogenic human liver fluke, Opisthorchis viverrini. BMC Genomics.

[25] Sripa B, Kaewkes S (2000). Relationship between parasite-specific antibody responses and intensity of Opisthorchis viverrini infection in hamsters. Parasite Immunol.

[26] Sripa B, Kaewkes S (2000). Localisation of parasite antigens and inflammatory responses in experimental opisthorchiasis. International journal for parasitology.

[27] Young ND (2010). Unlocking the transcriptomes of two carcinogenic parasites, Clonorchis sinensis and Opisthorchis viverrini. PLoS neglected tropical diseases.

[28] Hall T (1999). BioEdit: a user friendly biological sequence alignment editor and analysis program for Windows 95/98/NT. Nucleic Acids Symposium Series.

[29] Altschul SF (1997). Gapped BLAST and PSI-BLAST: a new generation of protein database search programs. Nucleic Acids Res.

[30] Thompson JD, Higgins DG, Gibson TJ (1994). CLUSTAL W: improving the sensitivity of progressive multiple sequence alignment through sequence weighting, position-specific gap penalties and weight matrix choice. Nucleic Acids Res..

[31] Saitou N, Nei M (1987). The neighbor-joining method: a new method for reconstructing phylogenetic trees. Mol Biol Evol..

[32] Tamura K, Stecher G, Peterson D, Filipski A, Kumar S (2013). MEGA6: Molecular Evolutionary Genetics Analysis version 6.0. Molecular biology and evolution.

[33] Papatpremsiri A (2015). Suppression of Ov-grn-1 encoding granulin of Opisthorchis viverrini inhibits proliferation of biliary epithelial cells. Experimental parasitology.

[34] Schmittgen TD, Livak KJ (2008). Analyzing real-time PCR data by the comparative C(T) method. Nat Protoc.

