# Supplementary material for: Suppression of mRNAs encoding CD63 family tetraspanins from the carcinogenic liver fluke Opisthorchis viverrini results in distinct tegument phenotypes
Source: Sci Rep. 2017 Oct 30;7:14342. doi: 10.1038/s41598-017-13527-5 (PMC5662742; doi:10.1038/s41598-017-13527-5)

# Supplementary information

**Suppression of mRNAs encoding CD63 family tetraspanins from the carcinogenic liver fluke *Opisthorchis viverrini* results in distinct tegument phenotypes.**

Sujittra Chaiyadet[1], Watchara Krueajampa[1], Wiphawi Hipkaeo[2], Yada Plosan[2], Supawadee Piratae[3], Javier Sotillo[4], Michael Smout[4], Banchob Sripa[5], Paul J Brindley[6], Alex Loukas[4]*, Thewarach Laha[1]*

[1]Department of Parasitology, Faculty of Medicine, Khon Kaen University, Khon Kaen, Thailand.
[2]Electron microscopy Laboratory, Department of Anatomy, Faculty of Medicine, Khon Kaen University, Khon Kaen, Thailand.
[3]Office of Academic Affairs, Faculty of Veterinary Sciences, Mahasarakham University, Mahasarakham, Thailand.
[4]Centre for Biodiscovery and Molecular Development of Therapeutics, Australian Institute of Tropical Health and Medicine, James Cook University, Cairns, QLD, Australia.
[5]Department of Pathology, Faculty of Medicine, Khon Kaen University, Khon Kaen, Thailand.
[6]Department of Microbiology, Immunology and Tropical Medicine, and Research Center for Neglected Diseases of Poverty, School of Medicine & Health Sciences, George Washington University, Washington, DC 20037, USA
*Correspondence: Thewarach Laha, PhD, Department of Parasitology, Faculty of Medicine, Khon Kaen University 40002, Thailand. (thewa_la@kku.ac.th); Alex Loukas, PhD, Australian Institute of Tropical Health and Medicine, James Cook University, Cairns, McGregor Rd, Smithfield 4878, QLD, Australia. (alex.loukas@jcu.edu.au)

**Supplementary Figures**

**Supplementary Fig. s1.**

Multiple alignment of deduced amino acid sequences from the CD63 tetraspanin family.

The alignment was constructed with homologous proteins derived from GenBank database, including *Opisthorchis viverrini* TSP-2 (AFY24238.1); *O. viverrini* TSP-3 (AFY24239.1); *Clonorchis sinensis Cs*-GAA50199.1; *Schistosoma mansoni* TSP-2 (AAN17276.1); *Schistosoma japonicum Sj*-CAX70616.1; *Homo sapiens* CD63 antigen (NP_001771.1).

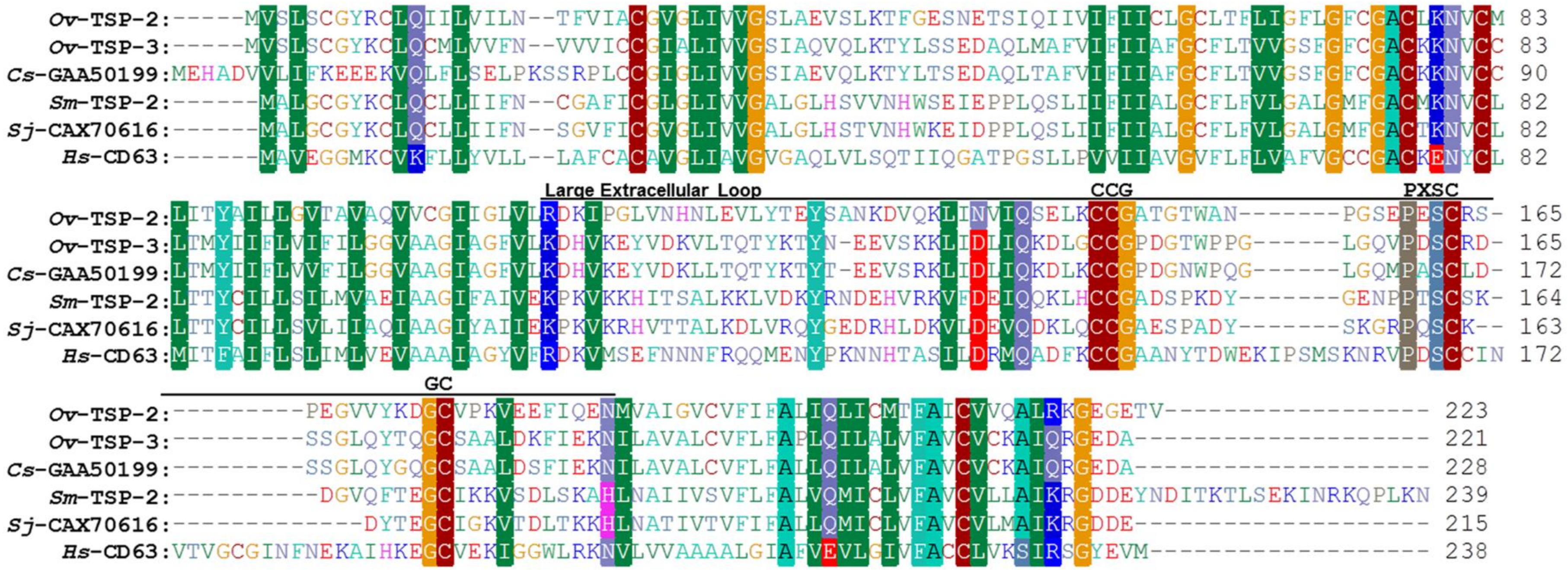

**Supplementary fig. S2.** Coomassie stain SDS-PAGE gels. The six full-length gels show lanes loaded with 23 sequentially eluted fractions from Ni-NTA affinity chromatograhy. Recombinant *Ov*-TSP-2 and -TSP-3 shown on the left and right, respectively. Images of the gels were electronically recorded, in tif format, using the Gel Doc XP with Image Lab software (BioRad) using the system's default standard contrast auto parameters.

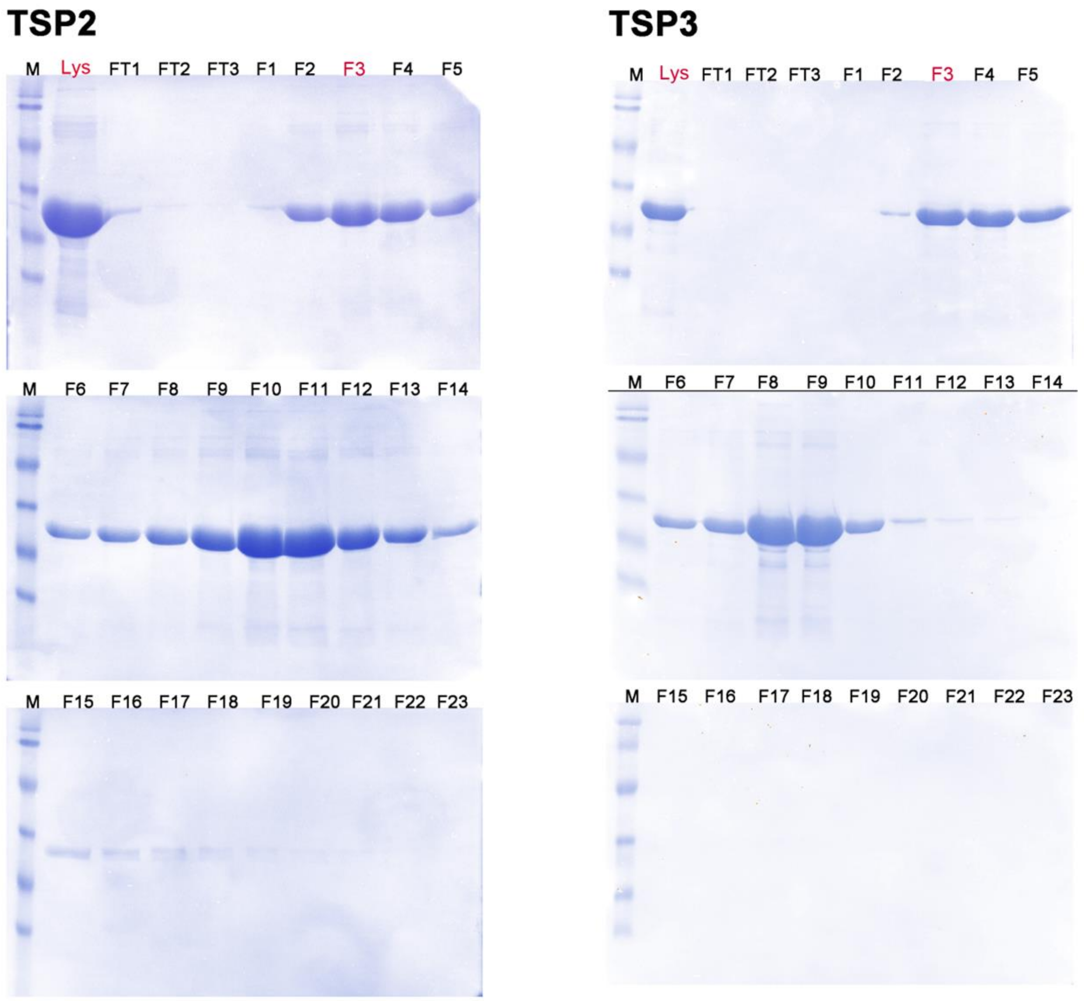

**Supplementary fig. S3.** Full-length, ethidium bromide stained agarose gels, showing the lanes loaded with equal volume (10 µl/lane) of RT-PCR products. RT-PCR products detecting *Ov-tsp-2* and Ov-*tsp*-3 from developmental stages shown on the left and right, respectively. Molecular size standards are shown at the left lane of each gel. Images of the gels were electronically recorded, in tif format, using the Gel Doc XP with Image Lab software (BioRad) using the system's default standard contrast auto parameters. Juv, juvenile; Meta, metacercaria.

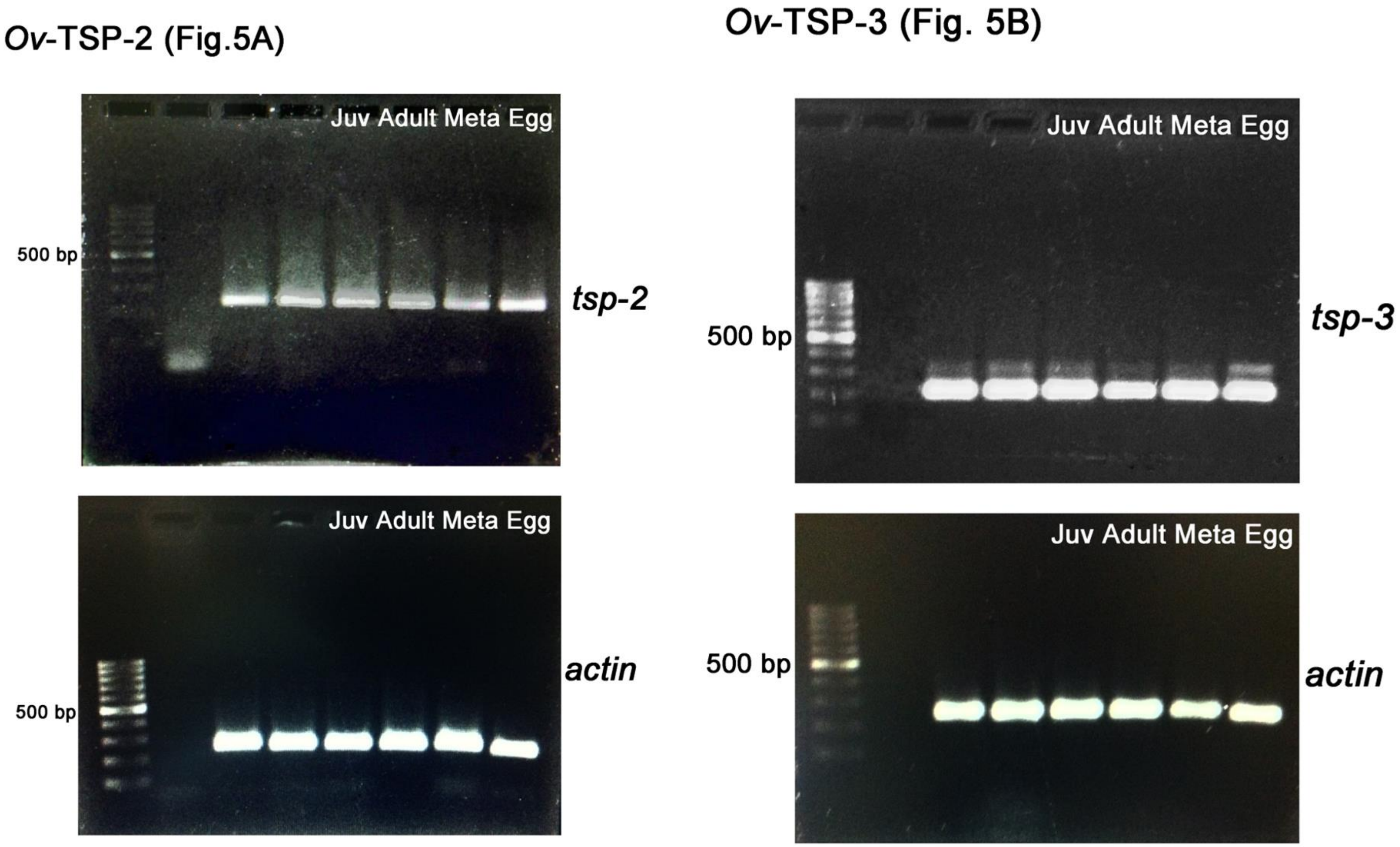

**Supplementary fig. S4.** Full length western blots to analyze soluble extracts of developmental stages of *O. viverrini* probed of with rabbit anti-*Ov*-TSP-2 (A) or rabbit anti-*Ov*-TSP-3 (B) sera. Intensity of reaction bands was quantified using densitometry.. Images of the membrane blots gels were electronically recorded, in tif format, using the Gel Doc XP with Image Lab software (BioRad) with the system's default standard contrast auto parameters.

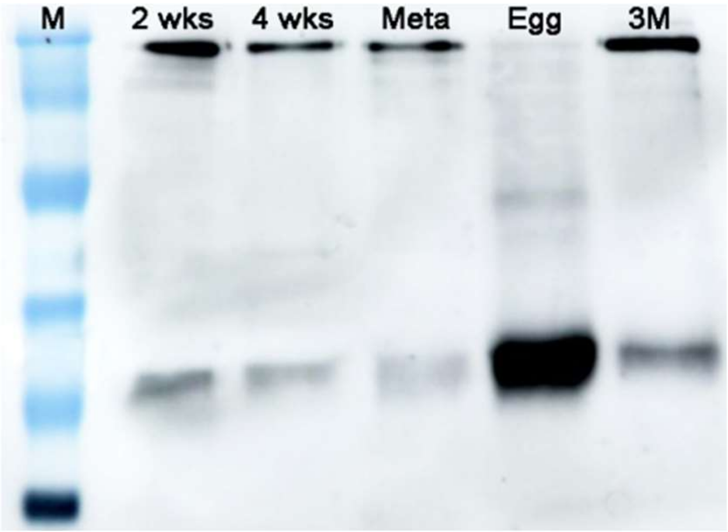


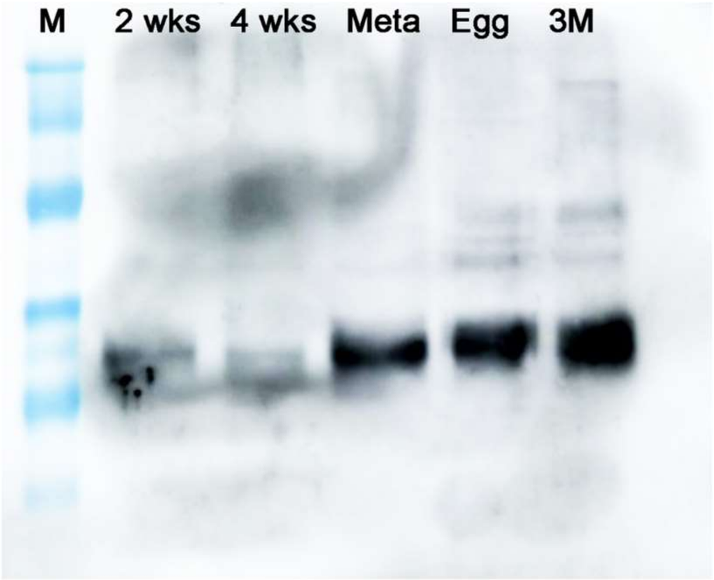

**Supplementary fig. S5** Full-length, ethidium bromide stained agarose gels, showing the lanes loaded with equal volume (10 µl/lane) of RT-PCR products. RT-PCR products detecting *Ov-tsp-2* and Ov-*tsp*-3 from the adult developmental stage of *O. viverrini* at increasing intervals (D1, day 1; D3, day 3, and so on) following RNA interference. Molecular size standards are shown at the left lane of each gel. Images of the gels were electronically recorded, in tif format, using the Gel Doc XP with Image Lab software (BioRad) using the system's default standard contrast auto parameters.

Fig.6A

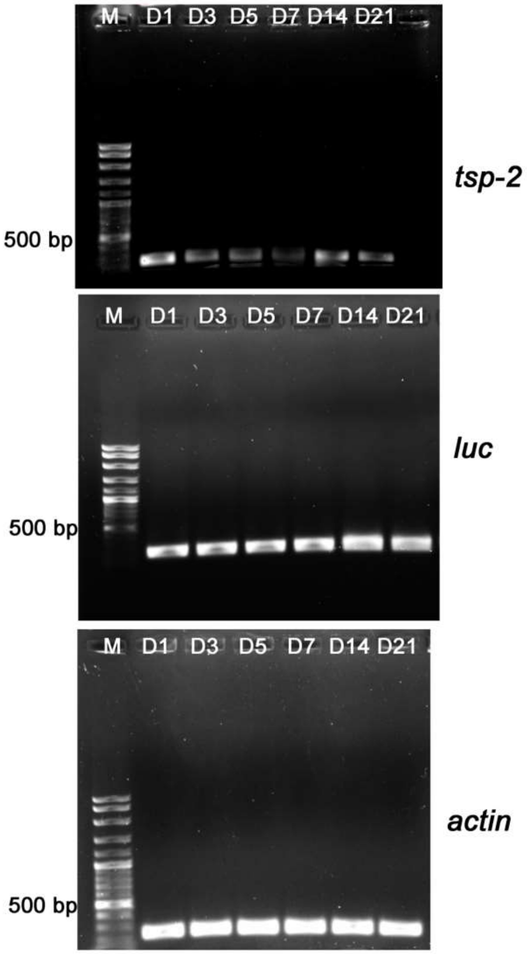


Fig. 6B

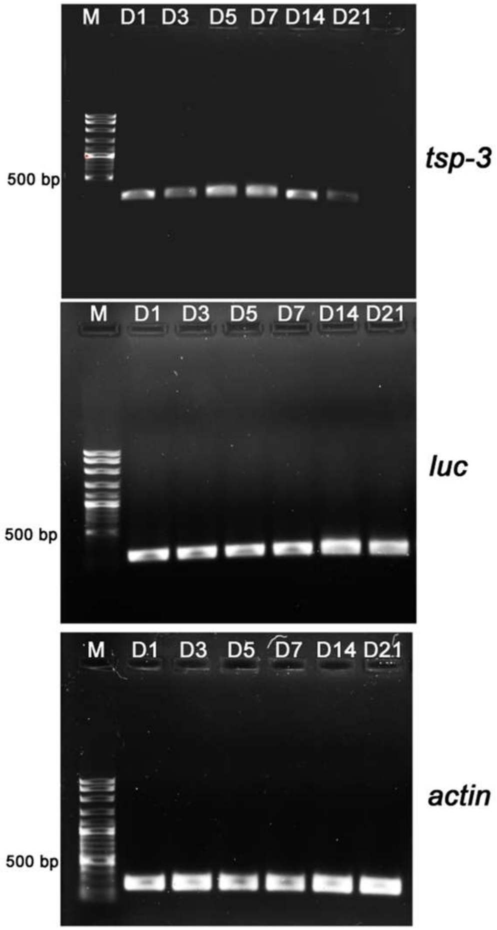

**Supplementary fig. S6** The full-length membranes of western blot analysis of soluble lysates of transfected *O. viverrini* probed of with rabbit anti-*Ov*-TSP-2 (A) or rabbit anti-*Ov*-TSP-3 (B). Western blot band intensity was quantified using densitometry. Images of the gels were electronically recorded, in tif format, using the Gel Doc XP with Image Lab software (BioRad) using the system's default standard contrast auto parameters.

**Fig. 6C**

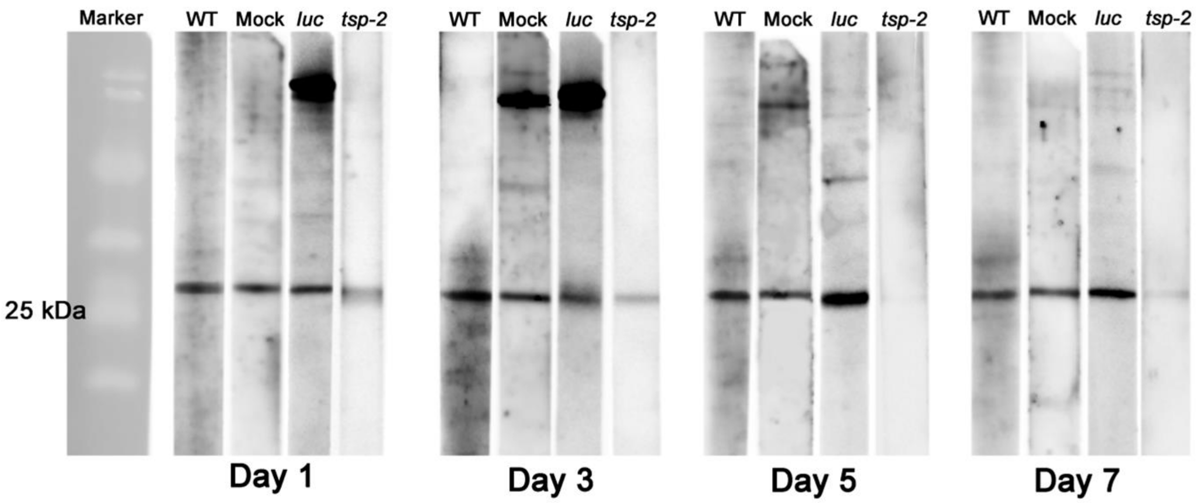


**Fig. 6D**

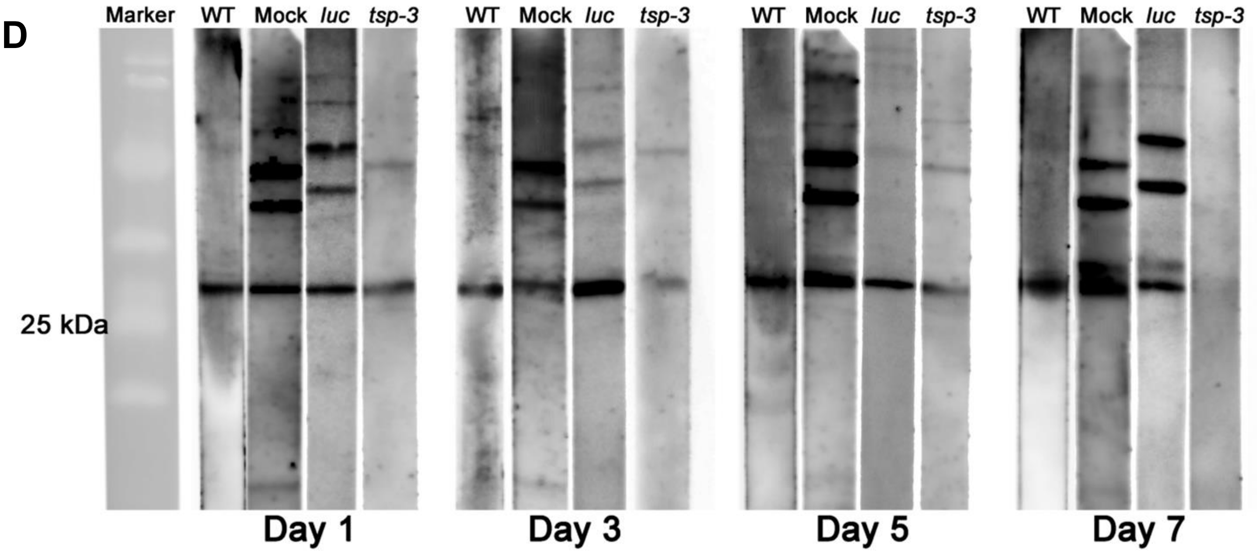

Supplement: Supplementary file 1 — Supplementary figures [file 41598_2017_13527_MOESM1_ESM.pdf]
